# Editorial: Direct and Inverse Comorbidities Between Complex Disorders

**DOI:** 10.3389/fphys.2016.00117

**Published:** 2016-03-30

**Authors:** Rafael Tabarés-Seisdedos, Anaïs Baudot

**Affiliations:** ^1^Department of Medicine, University of Valencia/CIBERSAM and INCLIVA Health Research InstituteValencia, Spain; ^2^Aix Marseille Université, CNRS, Centrale Marseille, I2M UMR 7373Marseille, France

**Keywords:** comorbidity, multimorbidity, complex diseases, OMICS data, medicine

**Comorbidity** and **multimorbidity**, defined as the presence of more than one disease in individuals, have emerged as a major challenge in the last decade (Valderas et al., 2009). Indeed, researchers, health professionals, healthcare managers and policy makers, and patients and citizens are lagging behind considering the comorbidity scenario, as illustrated by the paucity of documentation concerning interventions in people with multiple conditions (Smith et al., 2012). There is a clear need to better understand disease-disease relationships, in order to better organize and provide care, but also to develop appropriate research models. We can first characterize **direct multimorbidity** (higher-than-expected co-occurrence of diseases) and **inverse multimorbidity** (lower-than-expected co-occurrence of diseases). Examples of such disease associations include for instance diabetes mellitus associated with increased co-occurrence of depression (Rotella and Mannucci, 2013), or Alzheimer's disease associated with decreased co-occurrence of cancers (Catalá-López et al., 2014).

The seven articles published in this Research Topic “Direct and Inverse Comorbidities between Complex Disorders” are a step toward better understanding the complex relationships between diseases. Indeed, a large variety of approaches were assembled, spanning research fields such as bioinformatics to epidemiology, and diseases such as Alzheimer's or Malaria.

At the genomic level, Forés-Martos et al. studied the inverse comorbidity between Down syndrome and some tumors. Regions frequently deleted in tumors are expected to contain tumor-suppressor genes. The authors hypothesized that these tumor-deleted regions in chromosome 21, having an extra-copy in individuals with Down syndrome, could be protective against cancer. Zanzoni et al. studied extremely multifunctional proteins, which encompass proteins functioning in unrelated biological processes (i.e., moonlighting). The authors observed that these extremely multifunctional proteins are associated to more diseases, to complex rather than rare diseases, and to diseases with distant phenotypes. Overall, their results suggest that protein pleiotropy could be one of the factors of disease comorbidity. At the cellular scale too, Demetrius et al. discussed the amyloid and inverse Warburg hypotheses in the hallmark of Alzheimer's disease. They opposed these two competing models in the particular context of cancer inverse comorbidity.

At the level of individuals and populations, Roitmann et al. mined thoroughly patient medical records to fetch adverse events, and developed a network-based approach to cluster patients with similar profiles. Nudelman et al. revisited the previously described inverse comorbidity between Alzheimer's disease and cancer, thanks to a detailed analysis of different neurocognitive status and age of onset of Alzheimer's patients. Also working on the topic of neurocognitive comorbidities, Balanzà-Martinez et al. studied the correlations between bipolar and alcohol use disorders. Finally, Faure reviewed the current and historical literature describing direct and inverse relationships between malaria infections and other widespread diseases.

What general messages might readers take away? The traditional classification of diseases in watertight categories, and the management of patients as “biomedical” rather than “biopsychosocial” definitely exerted a powerful gravitational effect on medical practice, education and investigation. The reductionist approach, focusing on isolated diagnosis, disease, biological processes, or genes has limited progress by ignoring the relationships and comorbidities of diseases. Contrarily, the papers gathered in this Research Topic show that it is time to think outside of the box, and to search for clues in unexpected places.

Many factors are suspected to generate dependencies between diseases. They are linked to the environment, lifestyle, drug treatments or genetic factors, and more probably, a combination thereof. For instance, the same genes or proteins can be involved in different diseases. And as the biological macromolecules do not act isolated, but rather interact to perform their cellular functions, the intricate biological processes could also be one of the bases of molecular comorbidities. In this context, the molecular and cellular interactions between macromolecules depicted by -omics tools offer an unprecedented opportunity to understand the etiology and pathophysiology of disease comorbidities. At the level of individuals and populations, the data era also enables mining clinical practice via electronic health records and medical databases, and maybe in a close future personal health devices. Overall, the multimorbidity vision could help us refine current nosology of diseases and lead eventually to the development of a new transdisciplinary approach and a new model of medicine, which may in turn provide new therapeutic strategies.

## Author contributions

RT and AB were both co-editors of the Research Topic entitled “Direct and Inverse Comorbidities between Complex Disorders,” and each edited a subset of the articles. The editorial has been written jointly.

### Conflict of interest statement

The authors declare that the research was conducted in the absence of any commercial or financial relationships that could be construed as a potential conflict of interest.
